# A Pilot Retrospective Study on the Effect of Bone Grafting after Wisdom Teeth Extraction

**DOI:** 10.3390/ma14112844

**Published:** 2021-05-26

**Authors:** Luigi Canullo, Giampiero Rossi-Fedele, Francesca Camodeca, Maria Menini, Paolo Pesce

**Affiliations:** 1 Department of Periodontology, University of Bern, 3000 Bern, Switzerland; lugicanullo@yahoo.com; 2 Adelaide Dental School, The University of Adelaide, Adelaide, SA 5000, Australia; giampiero.rossi-fedele@adelaide.edu.au; 3 Private Practice, 00198 Rome, Italy; camodeca26@gmail.com; 4 Department of Surgical Sciences and Integrated Diagnostics, University of Genoa, 16100 Genoa, Italy; maria.menini@unige.it

**Keywords:** bone regeneration, third molar, tooth extraction, alveolar bone loss

## Abstract

This study aimed to retrospectively investigate the effect of bone graft after extraction of wisdom teeth impacting with the distal aspect of the second molar, on soft tissue wound healing, bone loss, and periodontal parameters. Sixteen patients treated an for impacted mandibular wisdom tooth at least one year ago were re-called (18 teeth). Dental panoramic tomography and periodontal parameters were assessed. A graft material was used to fill the post-extractive sockets in the test group (GUIDOR easy-graft CRYSTAL), whereas in the control group, the socket was filled using a collagen sponge and blood clot (Hemocollagene, Septodont, Matarò, Spain). The radiographic bone loss was measured at the distal aspect of the second molar. The Wilcoxon singed-rank test for paired data was performed to evaluate statistical differences. In the test group, only two cases out of nine showed bone loss, with an average of 0.55 ± 1.30 mm. Conversely, in the control group, five teeth out of nine showed bone resorption with an average of 1.22 ± 1.30 mm. However, the differences were not statistically significant. Periodontal parameters at the second molar demonstrated similar behavior between the test and control groups. Soft tissue healing complications were lower in the grafted compared to the comparator sites without reaching statistical significance. Within the limitations of the present study, no difference was found between the two groups.

## 1. Introduction

Third molar (M3) extraction is one of the most frequent surgical interventions in everyday dental practice [1], and wisdom teeth are the teeth with the highest percentage of impaction, especially the mandibular ones [2]. The prevalence of impaction ranges from 66% to 77% [3,4], and 68.5% of the impacted wisdom teeth are in close contact with the nearby root of the mandibular second molars (M2) [3].

Surgical removal of third molars has been associated with the risk of periodontal pockets development and bone loss on the distal aspect of the adjacent second molar [3,5,6,7,8], which can negatively affect the long-term prognosis of M2. Kugelberg et al. found that 43.3% of second molars showed pocket depth (PD) greater than 7 mm and 32.1% exhibited bone loss exceeding 4 mm, after two-year follow-up following adjacent third molar extraction [5]. Due to these risks, the indication for third molar removal is the subject of controversy when considering the benefits and risks of the procedure, though the American Association of Oral and Maxillofacial Surgeons still suggests a prophylactic extraction to prevent the development of pathological alterations around third or adjacent second molars [9].

The development of periodontal complications at the distal aspect of the M2 has been associated with three important risk factors, such as: preexisting periodontal pocket (attachment levels > 3 mm), subjects age (age > 26 years), and the relationship of proximity and inclination of the M3 in relation to the M2 (horizontal or mesioangular impactions) [7,10].

Different procedures have been proposed to reduce the periodontal risks of M2, including adjunctive root planning of the distal aspect of M2, various access flap designs, soft-tissue suturing, “orthodontic extraction” technique [11,12,13,14], regenerative procedures, and bone grafting techniques [2,15,16]. Osborne et al. showed no influence on the probing depth reduction or reattachment of gingival tissues if root planning of the distal root of M2 was performed [17]. Similarly, limited or no benefit was observed with different access flap designs used in the extraction procedure [18,19,20]. As of today, a clinically significant improvement in attachment levels has not been found in most of the randomized clinical trials evaluating various reconstructive techniques, such as autogenous bone, bone substitutes (i.e., demineralized bone powder (DBP)) [21,22,23], guided-tissue regeneration (GTR) [22,24], soft-tissue procedures, or platelet rich-plasm [25].

It remains uncertain whether any of the proposed treatment approaches result in better periodontal outcomes at the distal surface of the M2 [26]. Despite this, it seems that the use of a graft material in the alveolus consents to reach a soft tissue seal, to create a barrier to reduce the risk of bacterial contamination of the clot, to rapidly restore the blood circulation, and therefore to accelerate the healing process [22].

Amongst the commercially available graft materials, GUIDOR easy-graft (Sunstar Suisse SA, Etoy, Switzerland) is a malleable and adhesive biomaterial (Phase-pure ß-tricalcium phosphate), applicable directly from the syringe, which, in contact with the blood, sets in a few minutes, forming a porous bridge that adapts to the morphology of the defect, thus offering ideal coagulum stability and improving the healing of hard and soft tissues.

The aim of this study was to evaluate if the use of a graft material (GUIDOR easy-graft CRYSTAL) could improve the periodontal conditions after wisdom tooth surgical removal compared to the use of a collagen sponge and blood clot. The tested null hypothesis was that no differences in periodontal parameters were present among the two groups one year after the extractions.

## 2. Materials and Methods

### 2.1. Study Design and Patient Selection

The present study was performed following the principles outlined in the World Medical Association Declaration of Helsinki (version 9 July 2018) on experimentation involving human subjects and the additional requirements of Italian law, and approved by the Ethical Committee of the University of Genoa (2021/38).

A convenience sample of patients treated 12 months earlier for impacted mandibular wisdom tooth extraction was included.

Patients were included if a pre-extraction panoramic tomography exam was available and if periodontal indexes were collected before surgery.

**Figure 1:**
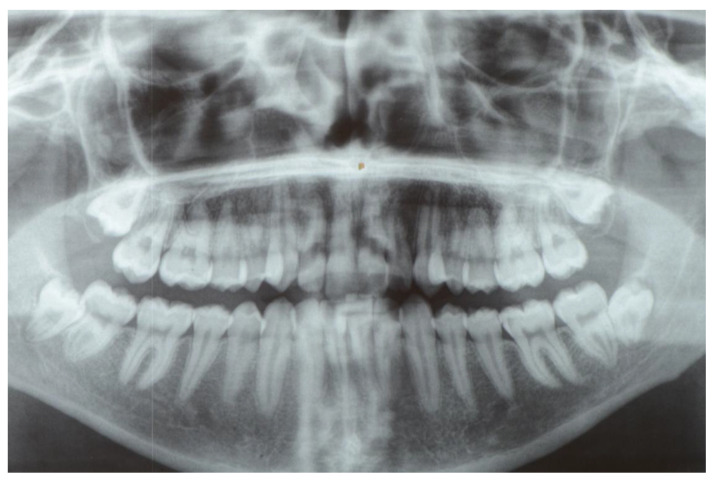
Pre-operative panoramic radiographic exam showing the impacting wisdom teeth.

Panoramic tomography was used to evaluate and classify the tooth inclusion, as proposed by Winter [27] (Figure 1).

All patients were in general good health. They were informed about the procedure and required to sign a consent form. Exclusion criteria are summarized in Table 1.

### 2.2. Preoperative and Postoperative Medication

One week before the surgical procedure, a full-mouth professional prophylaxis appointment was performed. Periodontal parameters (bleeding on probing (BoP), pocket probing depth (PPD), periodontal index (Pi)) were taken at the distal aspect of the second molar (T0). Patients were instructed to use 1 g penicillin clavulanate 1 day before surgery and continued with 2 g per day for 6 days. Just before surgery, patients underwent a 5 min mouth rinse with 0.2% chlorhexidine gluconate (GUM Paroex 0.2%, Sunstar Suisse S.A., Etoy, Switzerland).

### 2.3. Surgical Technique

After anesthesia, a sulcular incision was performed, starting mesially to the first molar to the distal surface of the second molar, and a relieving incision was performed distally at the ascending ramus but not mesially; thus, a full-thickness flap was elevated to expose the third molar (i.e., modified envelope flap). Once the tooth was extracted, the exposed root was meticulously cleaned using ultrasounds and a chlorhexidine gel (Corsodyl Gel, GLAXOSMITHKLINE C.HEALTH.SpA, London, UK) was placed for 60 s.

Patients included were divided into two groups.

**Figure 2:**
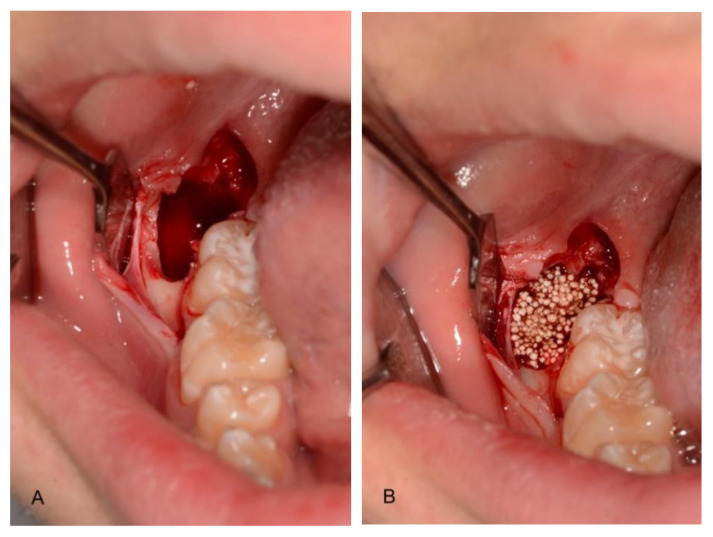
Post-extraction site (**A**) filled with the graft material (**B**) (test group).

In the test group, the socket was filled with a synthetic bone (GUIDOR easy-graft CRYSTAL, Etoy, Switzerland) (Figure 2, Table 2).

The graft material, applicable directly from the syringe, was placed at the distal aspect of the defect and against the medial aspect of the post-extraction cavity. The graft material was accurately condensed at each stage. No membrane was used to cover the defects.

**Figure 3:**
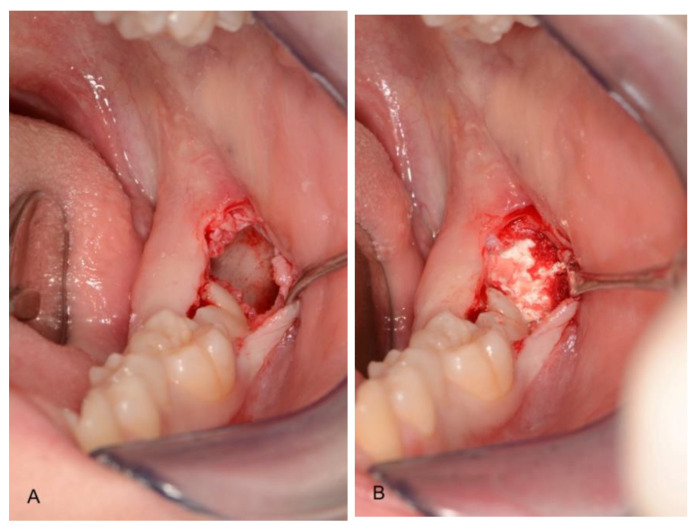
Post-extraction site (**A**) filled with blood and collagen (**B**) (control group).

In the control group, the socket was filled by a collagen sponge and blood (Hemocollagene, Septodont, Matarò, Spain) (Figure 3).

Before suturing, a periosteal releasing incision was performed to allow soft tissue closure by first intention.

The oral mucosa was then sutured with 5.0 non-resorbable (Polynil, Sweden & Martina, Padua, Italy) using interrupted sutures.

### 2.4. Postoperative Treatment

Post-operative anti-inflammatory medications and antiseptic mouth rinse were prescribed. At the time of sutures removal (14 days post-op), clinical and surgical postoperative complications were assessed.

### 2.5. Follow-Up Evaluation

**Figure 4:**
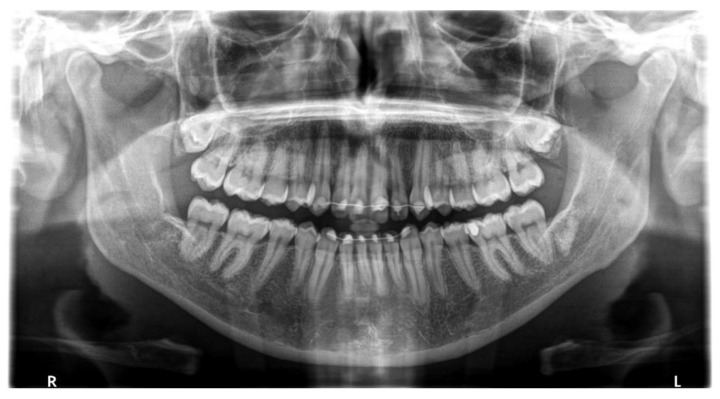
Post-operative panoramic radiographic exam showing bone regeneration in both control and test sites.

**Figure 5:**
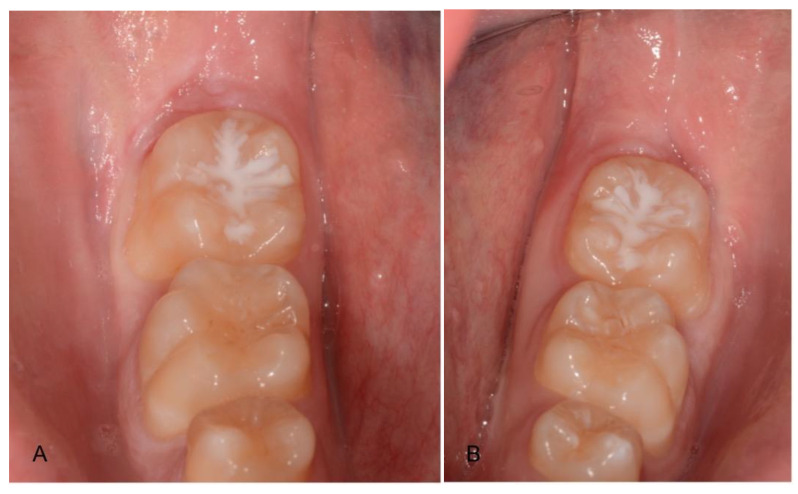
Post-extraction healing of test (**A**) and control (**B**) groups.

Patients underwent a digital dental panoramic tomography exam for postoperative evaluation after one year (Figure 4 and Figure 5).

In the same appointment, periodontal parameters (BoP, PPD, PI) were taken at the distal aspect of the second molar (T1).

### 2.6. Complications

Any biological (pain, swelling, suppuration) or surgical (graft material exposure, flap second intention healing) complications were collected from the patient files.

### 2.7. Radiographic Evaluation

The areas of interest were evaluated with a computerized measuring technique applied to digital panoramic radiographs (12-month follow-up). The same machine was used to perform both radiographs. Using the radiological software (CS Imaging 7—Carestream Dental, Rochester, NY, USA), bone loss at the second molar distal aspect was measured. Cement/enamel junction (CEJ) was used as the coronal reference. Bone loss was measured as the distance between CEJ and the first bone in contact with the root.

All radiological and periodontal measurements were conducted and collected by the same trained independent examiner, who was blinded to sample group allocation.

### 2.8. Sample Size Calculation

Sample size calculation was performed using the Statulator (http://statulator.com/SampleSize/ss2PM.html (accessed on 2 February 2021)). The sample size calculation was based on radiographic measures of mean bone loss on the second molar from CEJ, 1 year after third molar extraction [28]. The group compared extraction sites treated by GTR (test) or without any treatment (control). The group reported a 4.03 ± 1.08 mm loss for the control and 2.70 ± 0.37 mm loss for the GTR group. For assessing the sample size, an expected mean difference of 1.27 mm and a standard deviation of 0.95 was calculated using a correlation factor of 0.5 according to the Cochran handbook, Section 16.1.3.2 (SDchange = SQRT (SD^2^baseline + SD^2^final − (2 × correlation × SDbaseline × SDfinal))).

The significance level (alpha) was set to 5% (two-sided), and the power to 90%. The null hypothesis was set for equivalence of the test compared to the control group.

A total of 16 patients indicated for extraction of impacted third molars were deemed necessary.

### 2.9. Statistical Analysis

Descriptive statistics including mean values and standard deviation were used to describe the behavior in terms of bone loss and periodontal parameters between the two treatments.

Due to the small sample sizes, a Wilcoxon singed-rank test for paired data was performed to calculate *p*-values for intergroup differences at different time points (*p* < 0.05). Moreover, to test the hypothesis for significance of intergroup differences on the main endpoints of bone loss and PPD changes over time (T1–T2), a Wilcoxon signed-rank test for paired samples was used to calculate *p*-values, and confidence intervals at 95% (CI95) of the differences were calculated.

## 3. Results

The study sample consisted of 18 lower third molars impacting on the distal aspect of the second molar, in 16 patients. As classified by Winter et al., 4 molars were horizontal, 4 vertical, and 10 mesioangular. The mean age at surgery was 19.5 years, with a range of 16 to 28 years. All patients (6 male, 3 female) presented according to the American Society of Anesthesiologists (ASA) physical status classification system, ASA 1 conditions, and were non-smokers. Patients were free from infections [29].

### Periodontal Parameters

Periodontal parameters demonstrated similar behavior between the test and control group. Regarding the control group, BoP was 0.89 ± 0.33 on average at T0 and 0.22 ± 0.44 at T1, while in the test group, BoP was 0.78 ± 0.44 on average at T0 and 0.22 ± 0.44 at T1. There was no statistical difference between the two groups at the one-year follow-up. There was a slight decrease in the PI in both groups, however, the differences were not significant, showing in the test group values of 0.67 ± 0.5 and 0.44 ± 0.53 respectively, at T0 and T1 (*p* = 0.41), while in the control group, 0.56 ± 0.53 and 0.44 ± 0.53, respectively (*p* = 0.71).

In the test group, the mean PPD was 4.00 ± 1.41 at T0, while in the control group, it was 4.56 ± 1.67.

After 12-month follow-up, PPD was significantly reduced by 1.33 ± 1.32 mm (*p* = 0.038) for the test and 1.44 ± 1.24 mm (*p* = 0.016) for the control group. However, the difference between the two groups was not statistically significant. In fact, in the treatment group, mean PPD was 2.66 ± 0.5, with no case greater than 3 mm, while in the control group, mean PPD was 3.11 ± 0.78 mm and three cases showed PPD greater than 3 mm, specifically 4 mm.

**Figure 6:**
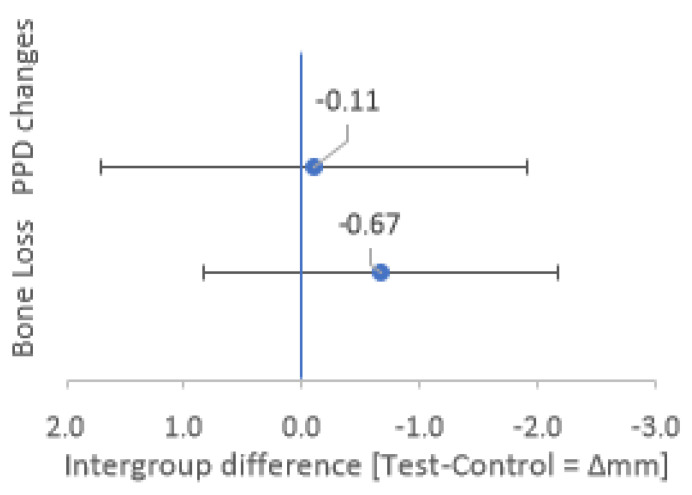
Intergroup differences for Bone Loss and PPD changes after 1 year. Mean intergroup differences (Test–Control) for Bone Loss and PPD changes after 1 year are represented by blue dots. The confidence intervals of 95% (CI95) for the intergroup differences are represented by the lower and upper whiskers.

The difference in PPD reduction between the groups (0.11 mm) was not significant (*p* = 0.336), as evidenced by the confidence interval including zero (Figure 6, CI95 Δ PPD: −1.7 to +1.97 mm).

Regarding complications (late healing), only two cases were found in the grafted group compared to four cases in the control group. No other complications were reported in the two groups.

In the test group, only two cases out of nine showed bone loss of 2 and 3 mm after one year, leading to a mean bone loss of 0.55 ± 1.30 mm. Conversely, in the control group, five teeth out of nine showed bone resorption, leading to a mean bone loss of 1.22 ± 1.30 mm after one year. However, the difference between the groups (0.67 mm) was not statistically significant (*p* = 0.34), as evidenced by the confidence interval of 95% including zero (Figure 6, CI95 Δ Bone Loss: −0.83 to +2.16 mm).

## 4. Discussion

The continuous challenge for clinicians has always been to optimize patient comfort and outcomes after impacted mandibular M3 extraction [30]. The principal risk after surgical management of mandibular M3 is to develop or have a persistent periodontal pocket and bone loss on the distal aspect of the mandibular M2 [3,21,22,31].

Therefore, the purpose of this retrospective study was to test if a bone graft could promote increased healing of soft and hard tissues after the extraction of a wisdom tooth impacting with the distal aspect of the second molar [31]. In particular, an in situ hard-setting synthetic bone graft material has been used as a scaffold onto which bone-forming cells, blood vessels, and other cells can migrate and form healthy new bone to repair the defect [32]. The results of this study confirmed the null hypothesis, suggesting that this regenerative therapy did not change the risk of developing or increasing a periodontal defect on the distal aspect of M2 when compared with the control group. Periodontal measurements showed similar behavior between the two groups. At the baseline, randomization was successful in distributing the mean periodontal parameters equally among the two groups [22].

To evaluate periodontal parameters, we performed only measurements at the distobuccal site of the second molar, because it was easily accessible and visible for measurements, to minimize random measurement errors and simplify the analysis of the results. Furthermore, in this way, it was possible to obtain preoperative measurements without having the physical interference of the M3, and it was considered as a significant site because it may be at risk for iatrogenic injury due to the osteotomy necessary for the extraction [22]. Our decision to select a single site is in agreement with what was carried out in studies by Dodson and Hassan, unlike other studies that have considered many more sites for measurements and have significantly increased the complexity of the analysis due to the problem of clustering correlated observations [22,33,34].

Three important factors that influence periodontal healing at the distal aspect of the M2 have been proposed: pre-existent periodontal defects, patient age, and inclination and degree of depth of the M3 [7,10].

In our study, the sample was formed by patients with a mean age of 19.5 and a pre-operative mean PPD of 4 ± 1.41 and 4.55 ± 1.66 respectively, in the test and control groups. Regarding the age, the patients were “young” considering that some previous studies have set the age of twenty-six as the threshold [24,28], and this may be a reason for the lack of significant differences in this study. A rationale for this, as proposed by Kugelberg and Church, could be that increasing age decreases the ability of the immune system to respond to bacterial plaque and this would explain the different periodontal responses between young and old patients [35,36].

In fact, most studies reported clinically (≤2 mm) and statistically non-significant values in young patients when GBR was compared with no treatment [22,28]. Although we did not find periodontal clinical differences between groups, the radiographic analysis showed less bone loss, measured from alveolar bone crest to CEJ, using synthetic bone grafts compared to blood clot [37].

When evaluating bone loss, we used the same type of radiography, thus obtaining a better comparison between results and understanding of bone remodeling during the healing process. Additionally, the use of X-ray imaging can bring about differential diagnosis in salivary pathology, such as calculus and others [38]. Instead, other studies did not use the same types of radiographs (panoramic or intraoral), or the same methods of measurement to evaluate radiographic outcomes [6,7,39,40,41], and this has led to a difficulty in comparing the results because it is necessary to consider the error of each type of radiographic technique [42,43]. In the present study, we chose to measure the bone loss one year after M3 removal with a digital panoramic, unlike the study of Faria et al., in which measurements were performed before surgery and at 3, 6, and 12 months after surgery [44].

Although no statistically or clinically significant differences between the two groups were found in our study in terms of bone loss, a trend to less bone resorption in the grafted sites could be observed compared to the spontaneously healed sites. Moreover, the use of graft material to fill the residual bone defect after extraction and the achievement of a primary closure allowed to reach a soft tissue seal. This supports the creation of a barrier to reduce the risk of bacterial contamination of the clot and to accelerate the healing of soft tissues [22]. This effect might have positively influenced the soft tissue healing of the M3 extractions sites, as fewer complications were seen in the grafted (2 out of 9) compared to the comparator sites (4 out of 9).

On the other hand, Debois et al. have compared primary and secondary closure techniques after extraction of impacted lower third molars, and they concluded that the best procedure was to heal the alveolus by secondary intention [45]. In fact, they observed that a secondary closure seemed to reduce postoperative edema, pain, and improve daily hygiene because the patient was able to clean more easily. However, these investigators did not evaluate patient comfort or resulting mucosal deformities, so it is not possible to establish the real greater clinical efficacy of this procedure.

Several studies report encouraging results on the use of graft materials after extraction of the third molar, such as Aimetti et al., who experimented guided bone regeneration by placing the membranes on the distal aspect of the lower M2, and one year after surgery, they noted statistical differences in the level of periodontal attachment and bone gain [28].

Moreover, the platelet-rich plasma (PRP) has been used successfully as a technique of bone regeneration after lower third molar removal. In particular, Sammartino et al., in 18 young patients, tested the use of a resorbable membrane of porcine origin in combination with PRP compared with PRP alone, and concluded that the two procedures showed the same clinical effect of osseous regeneration, and despite the use of the membrane in combination with platelet-rich plasma, preliminary signs of bone maturation were obtained, if analyzed from a histological point of view [25]. Furthermore, Hassan et al. researched the clinical effect of the combination of a xenograft and a membrane compared with spontaneous healing, in 28 patients aged 30 to 35 years old, and noted a statistical reduction in distal probing depth and a greater amount of bone in tested sites [32]. Nevertheless, there were no statistical differences between the two groups, although Dodson emphasized that there were patients who could benefit more from a bone regenerative technique and, in particular, those older than 26 years, who already have a periodontal defect on the adjacent second molar, or who have a mesioangular or horizontally impacted third molar [28]. Likewise, Karapataki et al. did not find statistical differences in the distal attachment level and probing depth, despite having placed resorbable or no resorbable membranes in distal intraosseous defects of at least 4 mm in 19 patients [25]. Another aspect that needs to be investigated is the role of the stem cells in the regeneration of extraction sockets [46].

The main limitations of the present study are represented by the retrospective design, the limited follow-up period, and the small sample size, plus the use of panoramic radiographs, as this imaging modality has limited sensitivity for the assessment of periodontal bone support.

Definitive conclusions cannot be drawn at the current stage. How much the differences found in the present study could impact the periodontal status of the second molar should be investigated in the long term and in a larger patient cohort. The short follow-up may allow only to speculate that the presence of a reduced pocket might be interpreted as a protective factor. The positive trend towards better maintenance of the bone healing and less soft tissue healing complications in grafted sites has to be further investigated in prospective studies.

## 5. Conclusions

Within the limitations of the present study, no statistically significant difference was found between groups. Both alternatives seem to be viable clinical options.

## Figures and Tables

**Table 1:** Exclusion criteria.

Exclusion Criteria
1. No impacted mandibular third molar extraction
2. No mesioangular/horizontal impacted wisdom tooth
3. Follow-up < 1 year

**Table 2:** Characteristics of the biomaterials.

Test	Control
Pre-filled syringe of polymer-coated granules together with a separate ampoule of polymer activator. This activator softens the polymer coating, creating a sticky surface. Granules are made of microporous calcium phosphates with pore sizes of 1 to 10 μm.	Hemostatic sponge with collagen of bovine origin, sterilized by beta radiations.

## Data Availability

The data presented in this study are available on request from the corresponding author.
